# Villagers’ Satisfaction Evaluation System of Rural Human Settlement Construction: Empirical Study of Suzhou in China’s Rapid Urbanization Area

**DOI:** 10.3390/ijerph191811472

**Published:** 2022-09-12

**Authors:** Lu Ye, Zihao Wu, Ting Wang, Kangle Ding, Yu Chen

**Affiliations:** 1 Center for Chinese Urbanization Studies, Soochow University, Suzhou 215123, China; 2 School of Architecture, Soochow University, Suzhou 215123, China; 3 Urban Planning Department, School of Civil Engineering and Architecture, Zhejiang University of Science and Technology, Hangzhou 310023, China; 4 Architecture Department, College of Landscape and Architecture, Zhejiang A&F University, Hangzhou 311300, China

**Keywords:** rural human settlement construction, satisfaction evaluation, villager-centered, factor analysis, rural revitalization

## Abstract

Continuous improvement of rural human settlements is a major realistic requirement of China’s economic and social development in the context of rural revitalization. Tracking and evaluating the phased progress of human settlement construction in stages represent important techniques for ensuring continual improvement. To improve the current objective data-based index system, this paper focuses on the villager-centered evaluation system at the village level. Factor analysis is used to screen the original data from the questionnaire and minimize the dimensions to synthesize common factors on the basis of empirical results. The main conclusions are as follows: (1) according to weight, the satisfaction evaluation system includes five common factors: living support facilities, nonagricultural industry income, agriculture production income, transportation infrastructure, and comprehensive ecological improvement. The results show that construction investment is beneficial, but not directly proportional to the villagers’ satisfaction. Actual improvement is not keeping up with the demand for public fitness, cultural, and recreational facilities. On the other hand, changes in villagers’ lifestyles may reduce the need for commercial facilities; (2) according to the evaluation model, the indicators can be classified into four categories on the basis of the weight assessment score, all of which can provide differentiated construction strategies to avoid duplication and inefficient resource waste. The survey data’s indicators of major differences between villages, such as public transportation and sanitation, need further discussion; (3) the gap between actual improvement actions and villagers’ needs gives an optimization path for rural construction. The experiences of sample villages in well-developed areas can be used as a model for policy formulation in other regions, and a long-term follow-up investigation should be included in future studies.

## 1. Introduction

The concept of human settlements was first proposed by the Greek scholar Doxiadis [1], marking the formation of the science of human settlements. Since human settlements are a fundamental issue related to human survival and development, the world organization has also been paying attention to the development of its theoretical research. In the 1970s and 1990s, the United Nations issued documents on human settlements. The Vancouver Declaration points out that human settlements are the collection of human society, including all social, material, organizational, spiritual, and cultural elements, covering cities, towns, or rural areas. They are composed of physical elements and the provision of supported service compositions [2]. It points out that efforts must be made to provide rural areas with the necessary infrastructure, public services, and employment opportunities [3]. In 2002, the United Nations established the Habitat Environment Program to promote the development of human settlements. The theme “Cities: Engine of Rural Development” [4] displayed on World Habitat Day pointed to the importance of coordinated development of urban and rural human settlements. In the report, it is particularly pointed out that China should promote research on rural human settlements.

The urban and rural human settlements in China are significantly different [5], and the differences between rural areas are also very unbalanced [6]. Problems such as rural environmental pollution [7], lagging development of public service facilities [8], and chaotic village construction [9] continue to intensify in the process of rapid industrialization and urbanization. These studies show a multidisciplinary research trend [10], and more and more scholars are beginning to cut in from the perspectives of urban and rural planning, geography, ecological environment, and sociology [11], researching the rural living environment. According to the discrimination of the main types of concepts of livability, environmental quality, quality of life, and sustainability, underlying conceptual models of livability were presented [12]. Ruth and Franklin explored the life course perspective on livability [13], and the indicator system for evaluating rural livability at the village level was put forward [14]. Many researchers have made great efforts in data acquisition, mainly from two types of data sources: attribute data from the statistics collected by the relevant state departments [15] and questionnaire data [16]. Natural environment data, such as meteorological data, terrain data, land-cover data, natural disaster data, public service facilities data, traffic data, population data, permanent resident population data, and social and economic data are widely adopted in the index system. In these evaluations, objective data as indicators of the evaluation are mainly applied to distinguish between the types of human settlements and regional differentiation characteristics [15,17,18,19,20]. In recent years, the evaluation of residents’ satisfaction with the living environment has attracted more attention. Studies have begun to focus on the value orientation of the user group as the starting point and attribution of the evaluation [21,22,23], as well as carrying out research on the construction of rural living environments from the perspective of villagers’ situations [24], analyzing the organic connection and interaction between the characteristics of rural human settlement elements [25,26], through the use of social–ecological systems, human–land relationship theory, and self-organization theory as frameworks to explore the influencing factors and processes of human settlement system evolution [27,28], as well as establishing a sociological index system [29,30,31].

It can be seen that the research on rural human settlements has gradually shifted from single-issue technical research on the natural environment to comprehensive research on the economy, society, and culture, and it has expanded to include the perspectives of multiple stakeholders. The research on the villager-centered evaluation of rural human settlement construction is not sufficient, especially microscopic research at the village scale. Therefore, this study aims to answer the following questions: How can a comprehensive evaluation index system be established to evaluate the staged results of the improvement of human settlements to the satisfaction of villagers? How should the findings of the village-scale rural human settlement assessment be incorporated into the next round of environmental planning and construction?

## 2. Understanding Rural Human Settlement Construction in China

As a traditional agricultural country, China has a more prominent duality between rural and urban areas [32]. China’s rural areas have long supported China’s rapid industrialization and urbanization [33]. As a reservoir to ensure social stability and resist urban economic crises (The Chinese countryside has been sacrificed in the process of China’s high-speed industrialization and urbanization. In the 30 years after the founding of the People’s Republic of China, the state took away 600 billion CNY from agriculture to finance the development of urban industrialization, which accumulated to the end of the 20th century and triggered the current crisis facing agriculture, the countryside, and farmers.), they have begun to receive great attention from the whole country. China has entered the stage of cities feeding back to the countryside, following a new urbanization path of urban and rural coordinated development [34]. Since 2004, the Party Central Committee’s “Document No. 1”, which decides the annual government priorities, has consecutively focused on rural issues for 18 years. However, in the early stages of the construction, many local governments, driven by the land economy, evolved the rural human settlement construction into large-scale demolition and construction, which led to the development of rural space. Irrational agglomeration has caused many rural problems, such as the loss of rural land [16,35,36], the demise of traditional settlements [37], the destruction of the ecological environment [38], and the disintegration of rural society [39]. In 2018, the General Office of the Central Committee of the Communist Party of China and the General Office of the State Council jointly issued the “Three-Year Action Plan for the Improvement of Rural Human Settlements (2018–2020)”, which established the implementation strategy for rural revitalization based on human settlements. In December of the same year, the Central Office and the State Office jointly issued the “Five-Year Action Plan for the Improvement of Rural Human Settlements (2021–2025)”. From “rectification” to “rectification and improvement”, the seemingly subtle changes in expression are not only a continuation of the policy achievements but also a new starting point for the establishment of a long-term mechanism for the construction of rural human settlements.

Therefore, in the new era of China’s rural development, it is more necessary to carry out scientific research on the construction of rural human settlements. The current study attempts to promote the timely evaluation of the phased human settlement improvement in developed rural areas. According to the human-centered theory of Doxiadis [40,41] and the five systems of Chinese scholar Wu [42], it is instructive to evaluate villager satisfaction from dimensions of infrastructure, quality of the living environment, public services, living standards, and environmental health, which also conform to the dimensions of the implementation path in China.

## 3. Materials and Methods

### 3.1. Study Area

**Figure 1:**
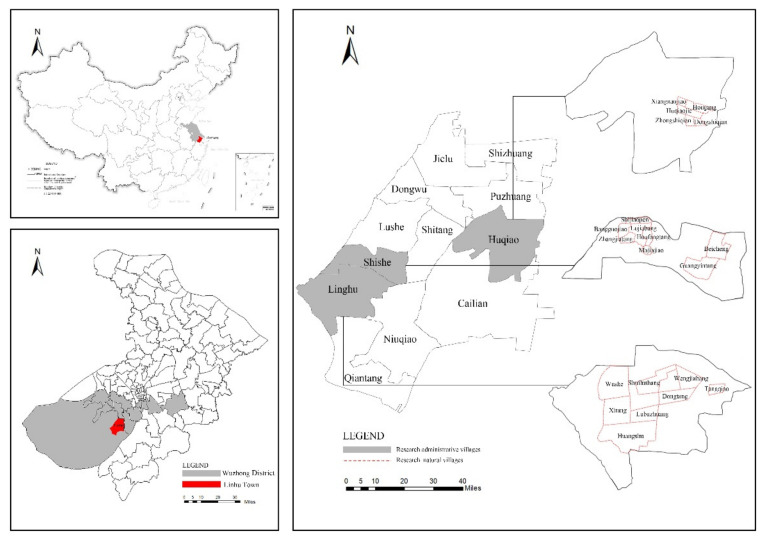
Location of Linhu Town in Suzhou.

Suzhou, a prefecture-level city in the Yangtze River Delta region, was chosen as the empirical study area. It is located in southeast Jiangsu Province (Figure 1). Suzhou’s urbanization rate was 77% in 2019. Its total GDP in 2020 was 2017 billion CNY, ranking sixth in China and representing the only prefecture-level city in the top 10 cities. The GDP per capita reached 179,174 CNY, after only Shenzhen in China. Suzhou is also known as oriental Venice, representing the abundant water resources and landscape of South Jiangsu Province. Linhu Town, located in the southwest of Suzhou and at the junction of the east and west sides of the Tai Lake, is one of the watertowns with distinguished regional features. Linhu Town covers 55.6 km^2^ and is made up of two communities, 12 administrative villages, and 173 natural villages. The majority of rural residents have changed their livelihoods from agriculture to secondary and tertiary industries. Since 2017, Linhu Town has been continuously improving the conditions of human settlements under the National Rural Revitalization Strategy and the Suzhou Rural Human Settlements Improvement Action, representing a demonstration area to promote the equalization of basic public services in urban and rural areas. Three administrative villages of Linhu Town, Shishe, Linghu, and Huqiao, were chosen to represent three distinct models of rural transformation: attractive demonstration villages, characteristic pastoral villages, and Suzhou Kangju characteristic villages (In the new rural construction, different standards of construction are applied according to the conditions and resources of each village. Suzhou has announced several types (rated beautiful demonstration village, characteristic pastoral village, Suzhou Kangju characteristic village, etc.) in stages and batches. The construction content differs according to the target; however, in general, in terms of construction investment standards, the beautiful demonstration village is the highest, while the Suzhou Kangju characteristic village is the lowest.). This study focuses on these three villages in Linhu Town because they have homogeneous geographical conditions and political contexts, allowing a comparison of the outcomes of different rural development approaches and how artificial construction affects rural human settlements.

### 3.2. Data Collection

On the basis of interviews with officials from Linhu Town’s rural construction department, 20 representative natural villages were chosen from the three administrative villages, including eight in Shishe Village (HFT, LJB, SJP, BGJ, ZJT, MJJ, BC, and GYT), seven in Linghu Village (DT, WJB, SLS, WS, LBZ, XT, and HS), and five in Huqiao Village (HQJ, HG, XNJ, DSQ, and ZSQ). Most of them have recently completed one round of rural human settlement improvement, with variation in the scale of construction.

First-hand research data were obtained during a 24-day field investigation in July and August 2020. Questionnaires were conducted through household interviews, and each questionnaire took at least 25 min to complete. A total of 521 questionnaires were collected, 496 of which were valid, with a valid rate of 95.2% and a valid coverage rate of 34.35% (Table 1). The gender ratio of men to women was 48:52, representing an overall balance. The sampling method in most households was systematic sampling, with <10% of participants recruited through a convenience sampling approach. The questionnaire included structured and open questions, divided into three parts: the first part consisted of basic information about the respondent and household (The first part includes individual age, gender, total family population, and population structure. Basic information such as homestead area, building floors, building area, construction age, and housing renovation content are included in the second part.); the second part consisted of 25 structured questions related to the theoretical model; the third part consisted of open questions, such as suggestions on the current individual’s willingness to participate in the construction and management process, as well as their intention to live in rural areas. Considering the fact that a relatively high proportion of respondents were elderly, in cases of illiteracy, the investigators explained each question face-to-face in the Suzhou dialect, allowed the respondents to reply, and then recorded the response on their behalf (At the beginning of the survey, all investigators were trained to understand the exact meaning of each question in the questionnaire, as well as how to understand the true evaluation of the elderly through some supplementary discourse when the respondents were unable to answer the questions directly.). In addition, this study also recorded notes of conversations with villagers and village officials about human settlements.

### 3.3. Evaluation and Analysis Methods

**Figure 2:**
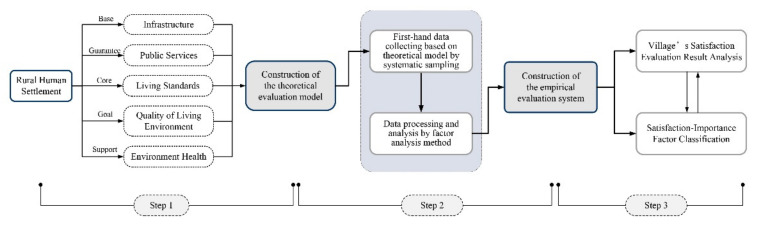
Framework on satisfaction evaluation of rural human settlements.

Three steps are involved in the method of evaluating rural human settlements: creating a theoretical model; collecting and processing data; developing an empirical evaluation system. To begin, the index in the theoretical model included as much information about rural human settlement construction as possible, according to pre-survey and interview data, as well as a literature review. Then, the study collected data on the theoretical model, before screening and reducing the dimension of the evaluation indicators using factor analysis (FA). The common factors obtained after dimension reduction can reflect the information of the original variables and simplify the irrelevant variables, thereby reducing the dimension of the variables and reinterpreting the original variable. The “dimension reduction/factor analysis” module was used for data analysis, with principal component analysis (PCA) as the extraction method. Lastly, the common factors were summarized to construct the empirical evaluation system, and a comprehensive evaluation result was also calculated on the basis of the system (Figure 2).

#### 3.3.1. Construction of the Theoretical Evaluation Model

According to the framework, this study constructed a theoretical evaluation model on villagers’ satisfaction in which the indicators reflect the connotation of this theoretical framework. As found through the literature review, human settlements mainly include five aspects, namely, infrastructure (i.e., supporting system), quality of living environment (i.e., living system), public services (i.e., human system), living standard (i.e., social system), and environmental health (i.e., natural system) [42]. Firstly, infrastructure is the foundation of rural human settlements, providing safe water, roads, and drainage [43]. Secondly, public services guarantee villagers’ participation in economic, political, and social activities [44]. Thirdly, living standards are the core of rural human settlements, which refer to the objective conditions related to residents’ income or consumption level [45]. Fourthly, based on the living standards, quality of life is a comprehensive reflection of the living and development requirements of residents [46]. Elevating residents’ quality of life in rural areas is the ultimate goal of improving rural human settlements. Fifthly, environmental health is an important factor to support the sustainable development of rural human settlements [47].

Under the guidance of the above principles, this study then adopted methods such as a literature review, expert consultation, and interviews to select indicators of the theoretical model. Firstly, the high-frequency and important factors reflecting the rural construction situation in the region were selected as evaluation indicators to build the basis of the theoretical model. Secondly, on the basis of theoretical research, expert consultation and interviews were used to solicit opinions and needs from multiple subjects such as experts, villagers, and village cadres, to optimize the theoretical model. The evaluation model contained a total of 25 indicators which could reflect the construction content and conform to the principle of the scientific method (Table 2). The indicators were scored using a Likert sample table.

#### 3.3.2. Data Processing and Analysis

The questionnaire used a Likert scale to investigate the villagers’ satisfaction with options of “very satisfied”, “moderately satisfied”, “general”, “moderately dissatisfied” and “very dissatisfied”, assigned a score of 5 to 1, respectively. For the questionnaire data, reliability analysis of the initial 25 variables was carried out first to judge whether the obtained data could be used for the next step. The results show that the Cronbach coefficient of the data was 0.911 (number of variable items = 25, *n* = 496), which is within the acceptable range, indicating that the questionnaire results were reliable.

Another important test is validity analysis, which tests the structural validity of the questionnaire indicators. The KMO and Bartlett tests were used to analyze the validity of the questionnaire data. The coefficient of the KMO test was 0.926, and the *p*-value of the Bartlett sphericity test was 0.000, both indicating the suitability of the data for FA.

The preliminary factor analysis results show that five common factors meeting the criteria are extracted, with a 56.358% cumulative variance contribution rate. The extraction standard was that the characteristic root should be greater than 1 [48], using the Varimax rotation method. The interpretation degree of the extracted common factor to each variable is shown by the common factor variance ratio, which ranges from 0 to 1. It can be seen that the common factor variance ratios of the nine variables were all less than 0.5 (Variables with a common factor variance ratio greater than 0.5 can be considered effective indicators in general). The reliability and validity analyses were repeated after removing these nine variables. The corrected Cronbach’s coefficient was 0.868 (number of variable items = 16, *n* = 496), indicating that the questionnaire results still had high reliability. Table 3 shows that the KMO test coefficient was 0.882, and the *p*-value of the Bartlett sphericity test was 0.000 (Table 3); thus, the data were still very suitable for FA.

#### 3.3.3. Extraction and Interpretation of Common Factors for Empirical Evaluation System

Factor analysis was repeated using the same method. The results showed that five common factors meeting the standard were extracted, with a cumulative variance contribution rate of 66.516%, which was increased compared with the result of 25 indicators, indicating that the elimination of indicators was relatively reasonable. Furthermore, the common factor variance ratio of all variables was greater than 0.5, indicating that the screened variables were suitable for inclusion in factor analysis as effective indicators (Table A1).

The screened evaluation indices had 16 variables in total, and FA was used to extract five common factors with characteristic values greater than 1 (Table A2). The initial contribution rates of the common factors were 35.521%, 9.655%, 7.426%, 7.263%, and 6.650%. To achieve a similar variance ratio explained by each common factor, and to bring the load coefficient closer to 1 or 0 to better explain the variables, the maximum variance rotation method (Varimax) was used. After rotation, the five common factors contributed to 17.179%, 15.598%, 12.899%, 11.323%, and 9.517% of the variation. The weight of each common factor in the total could be calculated using the variance contribution rate of each common factor after rotation (Table A3). The specific calculation method was as follows:

WF1 = 17.179%/66.516% = 0.2583.(1)

WF2 = 15.598%/66.516% = 0.2345.(2)

WF3 = 12.899%/66.516% = 0.1939.(3)

WF4 = 11.323%/66.516% = 0.1702.(4)

WF5 = 9.517%/66.516% = 0.1431.(5)

Using the weight of the common factor (Equations (1)–(5)) and the standardized variable score, the comprehensive score of the evaluation could be calculated as follows: composite scores = 0.2583F1 + 0.2345F2 + 0.1939F3 + 0.1702F4 + 0.1431F5.

From Table A3, it appears that the meanings of the five common factors extracted by FA were relatively clear according to the maximum variance method. Table A4 shows the rotated component score matrix obtained using the regression method. Each column of the component score coefficient vector in the table represents the linear combination coefficient of each index variable contained in each common factor. The score expression of the common factor is as follows: Fi = Zi1 × x1 + Zi2 × x2 + … + Zi16 × x16, where Zi1–Zi16 denote the variable coefficient vectors of the i factor, i.e.,

F1 = −0.058X1 − 0.040X2 + … − 0.133X16.(6)

F2 = −0.072X1 − 0.124X2 + … + 0.035X16.(7)

F3 = −0.031X1 − 0.064X2 + … − 0.041X16.(8)

F4 = 0.426X1 + 0.527X2 + … − 0.037X16.(9)

F5 = 0.105X1 + 0.022X2 + … + 0.619X16.(10)

## 4. Empirical Results and Analysis

### 4.1. Satisfaction Evaluation Model

#### 4.1.1. Common Factors of Model Based on Empirical Results

This study constructed a satisfaction evaluation model of rural human settlements based on empirical results using FA to screen the original data in the questionnaire and reduce the dimension to synthesize five common factors (F1–F5) to reinterpret the original information. The weights of each index in the evaluation model were computed on the basis of Table A3 and Table A4, and Equations (6)–(10) (Table 4).

1. Common factor F1 mainly included five indicators with a total value of 0.2583, among which fitness facilities and cultural entertainment facilities related to daily life accounted for the top two, followed by public activity venues and public toilets. Educational facilities had the lowest relative weight, unlike the top four indicators. F1 comprehensively reflects the villagers’ attention to the supporting facilities of daily life, representing a microscale life circle focusing on the villagers’ needs; hence, it was named the “life-supporting facilities factor”.
2. Common factor F2 included the impact of tourism, industrial development, and the village collective enterprise on revenues, as well as the impact of industrial development on their living environment. Among these four indicators, the weight of tourism development income was the highest with a value of 0.3557, far exceeding that of the traditional village collective enterprise income, which reflected the transformation of rural industrial development. F2 generally reflects the increasing concern of villagers about the impact of various developments in nonagricultural industry on their incomes; hence, it was called the “nonagricultural industry development factor”.
3. Common factor F3 included two indicators: agricultural planting conditions and facility improvement, as well as agricultural and forestry planting income, comprehensively reflecting villagers’ concerns about agricultural production conditions in their villages; hence, it was named the “agricultural production development factor”. The weights for both indicators were 0.5010 and 0.4990, respectively, indicating their similar importance, ranking among the first two indicators. Even in the highly urbanized and developed areas of China, villagers who no longer use agriculture as their main income still have a strong attachment to the land.
4. Common factor F4 included three indicators: parking lot, external public transport, and road facilities, with a total weight of 0.1702, comprehensively reflecting the priority of traffic infrastructure; hence, it was named the “traffic infrastructure factor”. The demand for motor vehicle parking lots ranked first, which also reflects that the transportation means of villagers were gradually being dominated by motor vehicles. Most of the villagers’ houses in the research area were built around 2000, and almost none of them were equipped with garages. This explains the demand for parking, which is usually concentrated at the entry or open space in the village. As a result, this highlights the demand for road width and flatness.
5. Common factor F5 included two indicators, air quality improvement and domestic pollutant remediation, with a weight of 0.1431, reflecting the concern for environmental governance; hence, it was named the “environmental governance factor”.

**Table 4:** Index system of satisfaction evaluation of rural human settlements based on empirical results.

Common Factor	Name	Weight	Index Factor			
			Number	Indicators	Common Factor Weight	Evaluation Target Weight
F1	Life-supporting facilities	0.2583	X6	Public fitness facilities	0.2735	0.0706
			X7	Cultural and recreational facilities	0.2469	0.0638
			X5	Public activity site and landscape reconstruction	0.2210	0.0571
			X4	Construction of public toilets	0.1646	0.0425
			X8	Educational facilities (training exchanges, schools, and societies)	0.0940	0.0243
F2	Nonagricultural industry development	0.2345	X12	Impact of rural tourism development on income	0.3557	0.0834
			X13	Impact of industrial development on the living environment	0.3195	0.0749
			X14	Impact of rural emerging industry development on income	0.2238	0.0525
			X11	Impact of village collective enterprises on income	0.1010	0.0237
F3	Agricultural production development	0.1939	X9	Improvement of planting conditions and agricultural auxiliary facilities	0.5010	0.0971
			X10	Impact of agricultural planting on income	0.4990	0.0968
F4	Traffic infrastructure	0.1702	X2	Motor vehicle parking	0.3748	0.0638
			X3	External public transport	0.3222	0.0548
			X1	Perfection and quality of village road regulation	0.3030	0.0516
F5	Environmental governance	0.1431	X16	Satisfaction with air quality	0.5454	0.0780
			X15	Satisfaction with domestic waste pollution control	0.4546	0.0651

#### 4.1.2. Characteristics of the Indicator System

From the information represented by the five common factors, it can be seen that the villagers pay the most attention to the improvement of daily living conditions, which is the aspect making the greatest difference between the lives of urban and rural areas in China. Secondly, the factor of nonagricultural industry development came in second place, showing the importance of the income of nonagricultural industries for villagers in eastern rural China. Furthermore, F3 shows the importance of agricultural production to the villagers, even in developed areas, also highlighting the embodiment of the bond between the villagers and the land on which they grow. The indicators of agricultural production were independent of the living standards of the target layer, forming a new common factor that again verified the deep-rooted reasons for the reluctance of most interviewed villagers to leave the countryside for the city. F4 reflects the increased need for traffic flow between urban and rural areas, as well as villagers’ newfound need for vehicle parking. The weight of F5 was relatively light because environmental restoration produced positive results in previous environmental regulations. Overall, the rural human settlement evaluation was extremely thorough, but it lacked prominent representative factors due to a weight difference of only 0.2583 to 0.1431.

Lastly, compared with the theoretical model, the nine indicators were not included in the final model of the villagers’ satisfaction. On the one hand, the differences between the two models reflect not only the interests of different subjects in the construction, but also the differences between the actual construction target and villagers’ needs, such as façade painting of houses, village architectural scene beautification, and the renovation of administrative facilities, which are not so important to the villagers. On the other hand, the indicators such as the lighting of village roads, the repair of revetments, the treatment of water pollution, and the treatment of production pollution were solved in the previous stage. In addition, the indicator of convenient commercial facilities in the theoretical model, as one of the important contents in the improvement, was eliminated in the factor analysis model, which showed that the villagers’ perception was relatively nonobvious. It was found that the advantages of online shopping platforms have gradually changed people’s ways of purchasing daily necessities according to the third part of the open interview. Therefore, the popularity of convenient express stations and private cars has increasingly changed the villagers’ lifestyles, as well as reduced the importance of commercial facilities in rural areas. This empirical model reflects the changing lives of villagers and has implications for future rural public service facility planning.

### 4.2. Satisfaction Evaluation Results

#### 4.2.1. Villager Satisfaction and Its Characteristics in Various Administrative Villages

The average comprehensive scores of each administrative village and its natural villages are shown in Table 5.

1. Huqiao Village: The evaluation score was the highest in terms of satisfaction with income from agricultural production, while that of transportation infrastructure was the lowest. The income from nonagricultural industries was lower than the average value, and satisfaction with life-supporting facilities and environmental governance were slightly higher than the average level. Among its natural villages, the greatest difference was reflected in the life-supporting facilities. ZSQ had the lowest satisfaction, mainly because of its geographical location, surrounded by other villages and relatively far from supporting facilities. In the interview, the respondents thought that environmental improvement mainly occurred near the main traffic roads and core areas and was ignored or simplified in the areas with poor accessibility. Therefore, construction should be avoided as an image project but rather a livelihood project that needs to pay attention to social justice.
2. Linghu Village: The satisfaction with transportation facilities was the highest, while that with life-supporting facilities, environmental governance, and nonagricultural income was lower than the average, and that with the income from agricultural production was slightly higher. The satisfaction difference among the seven natural villages was the largest, at 0.280 (HS) and −0.200 (WJB). Among them, WJB had the lowest satisfaction with life-supporting facilities, related to its relatively isolated geographical location, with no living facilities configured nearby in the renovation.
3. Shishe Village: In terms of life-supporting facilities, environmental improvement, and nonagricultural income, the satisfaction scores were the highest; satisfaction with the transportation infrastructure construction was slightly higher than the average level, ranking second, while that of agricultural income was the lowest, presenting a large gap with Huqiao Village and Linghu Village. From the radar map, it can be seen that the satisfaction with agricultural production development varied among its eight villages. The scores of BC and GYT were higher, while the other six natural villages ranked last in the 20 natural villages surveyed. This is related to the government’s relevant land policy. At the initial stage of regulation, the farmers’ cultivated land in these six villages was requisitioned for the construction of the Garden Expo Park located in the south of Shishe Village. Currently, the villagers live in the village, but they have completely lost their agricultural land.

**Table 5:** Common factors and comprehensive scores of each village.

Administrative Village	Natural Village	F1	F2	F3	F4	F5	Comprehensive Score
Huqiao Village	HQJ	0.128	−0.221	0.681	−0.600	−0.010	0.010
	HG	0.345	−0.309	0.693	−0.712	0.208	0.060
	XNJ	0.001	0.302	−0.031	−0.829	−0.037	−0.081
	DSQ	0.233	−0.018	0.318	−0.537	−0.074	0.016
	ZSQ	−0.640	−0.078	0.483	−0.737	0.375	−0.162
Subtotal		0.098	−0.106	0.477	−0.673	0.082	−0.010
Linghu Village	DT	−0.183	−0.544	0.502	0.327	−0.001	−0.022
	WJB	−0.990	−0.374	0.104	0.782	−0.066	−0.200
	SLS	−0.467	−0.168	−0.047	0.284	−0.071	−0.131
	WS	0.215	0.330	0.233	−0.005	−0.517	0.103
	LBZ	−0.535	−0.241	−0.003	0.548	−0.349	−0.152
	XT	−0.252	0.205	−0.107	−0.032	−0.335	−0.091
	HS	0.234	0.483	0.073	0.631	−0.109	0.280
Subtotal		−0.241	−0.026	0.119	0.339	−0.222	−0.019
Shishe Village	HFT	0.514	−0.055	−0.991	0.263	0.376	0.026
	LJB	0.446	0.334	−0.857	−0.017	0.396	0.081
	SJP	0.475	0.599	−0.815	0.347	0.279	0.204
	BGJ	−0.036	0.254	−0.563	0.309	0.443	0.057
	ZJT	0.345	−0.512	−0.392	−0.825	0.597	−0.162
	MJJ	0.375	0.224	−0.750	0.420	0.673	0.172
	BC	0.273	−0.060	0.132	−0.222	−0.696	−0.055
	GYT	−0.462	−0.036	0.021	0.296	0.051	−0.066
Subtotal		0.247	0.120	−0.538	0.075	0.234	0.034

Note: The common factors and comprehensive scores in the table were calculated after data standardization. A positive number indicates an above-average score, while a negative number indicates a below-average score.

**Figure 3:**
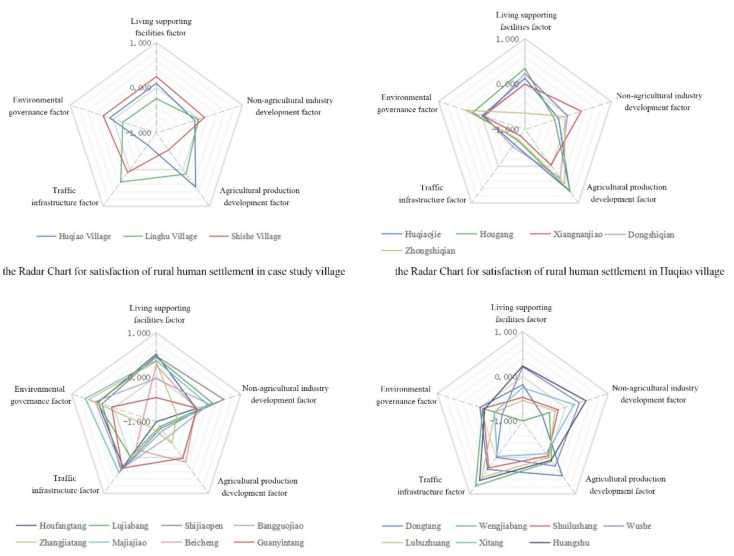
Radar chart for satisfaction of rural human settlement.

At the administrative village level, the comprehensive satisfaction score of Shishe Village was the highest, especially in three areas (F1, F2, and F5), while that of Huqiao Village and Linghu Village was lower than the average (Figure 3). This is positively related to the capital investment in construction, because the investment standard in Shishe Village is the highest. However, while Linghu Village has a higher construction standard than Huqiao Village, its contentment score was lower, indicating that the construction level is not proportionate to the villagers’ satisfaction. The difference among Huqiao’s natural villages was the smallest, with the standard value falling between 0.060 (HG) and −0.162 (ZSQ), while that in Linghu was the largest, ranging from 0.280 (HS) to −0.200 (WJB), which were also the highest and lowest scores among all villages. The satisfaction differences generated by the improvement in the village express the villagers’ demand for fairness on a microscale. Shishe Village’s lowest score for F3 illustrates the importance of agriculture and land for villagers, even in the high-investment villages of eastern rural China.

#### 4.2.2. Analysis of Relationship between Indicator Importance and Satisfaction Score

**Figure 4:**
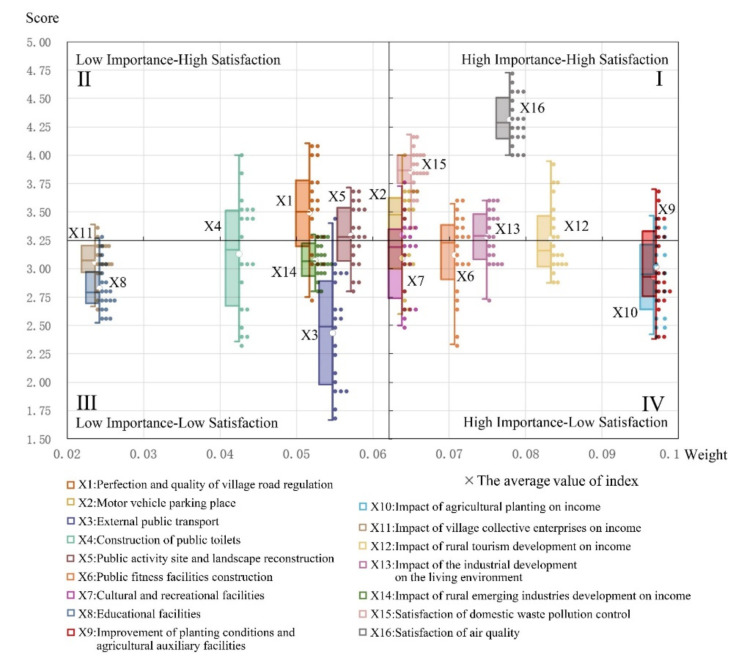
Evaluation of satisfaction and importance factor classification.

The relationship between the importance weight and the satisfaction score of each indicator is shown in Figure 4. On the basis of the average value of the index satisfaction score, the commonness of the survey area was obtained, and the results highlighted in four different quadrants.

The first was the high importance/high satisfaction quadrant, which included five indicators: air quality, domestic pollution, parking lot, industry’s impact factors on the living environment, and the income of nonagricultural industries. The corresponding contents were reasonably perfect in most villages, and the subsequent investment can be appropriately decreased by focusing on maintaining the achievements, allowing the successful conditions to be continually improved.

The second was the low importance/high satisfaction quadrant, including road facilities and activity sites in the village. In the sample villages, the villagers were satisfied with the content, and the feedback on the index was not very strong; thus, there is no need to invest too much in subsequent construction.

The third was the low importance/low satisfaction quadrant, comprising five indicators: public toilets, public transportation, income impact, village enterprises, and educational facilities. In the sample villages, the setting of such indicators was not perfect, and the feedback of villagers was not strong, indicating that the priority for the follow-up renovation of indicators is low. Indicators with low satisfaction but high importance, such as public transportation, should be included as a priority aim in this quadrant.

The fourth was the high importance/low satisfaction quadrant, including cultural entertainment, fitness facilities, planting conditions, and planting income. In the sample villages, villagers gave strong feedback on these indicators and expressed low satisfaction with the present conditions. There is extensive room for improvement, which should be made a priority in the next round of improvement.

It should be noted that the boxplot is also superimposed to convey the satisfaction score of each indicator, which reflects the situation in various natural villages. It can be seen that the distribution interval difference value (maximum and minimum) of most scores was between 0.5 and 1.5, highlighting that, although there were differences in villagers’ satisfaction in different natural villages, they were also in line with the situation reflected by the mean value. Among them, the difference in distribution between public transportation and public toilets was relatively wide, indicating that there were significant differences among natural villages. Therefore, further discussion is required on these two indicators, also suggesting that attention should be given to policy implementation by the village.

## 5. Discussion

### 5.1. Construction of a Villager-Centered Evaluation System for the Improvement of Rural Human Settlements in Various Regional Types

This study focused on the villagers’ satisfaction evaluations of the human settlement construction on the microscale in typical sample villages. This study attempted to avoid the inevitable influence of construction-related political objectives and other interests. For this purpose, factor analysis was adopted to screen the original data in the questionnaire and reduce the dimension to synthesize common factors to reinterpret the original information; accordingly, common factors were selected and weighted on the basis of this first-hand empirical data. Then, the villagers’ satisfaction evaluation model was built to reflect the needs of the villagers. Rural human settlement construction needs to give full play to the main role of villagers. The empirical study showed that the science of construction standards still needs to be improved, and the relationship with the principle of fairness needs to be further discussed. The single configuration of supporting service facilities makes it difficult to meet the needs of villagers’ daily lives. It is crucial to think about how to develop a scientific evaluation system that will meet the needs of the villagers for various areas because rural China has significant geographical and economic differences.

### 5.2. Guidance for the Next Round of Rural Construction for Rural Vitalization

Improving rural human settlements is a powerful starting point for achieving rural revitalization. Many policies and action plans have been issued in coordination at the national, provincial, and municipal levels, vigorously promoting the improvement of rural human settlements, which is a top-down government project carried out on a national scale. Through the whole process, from planning to design, and then to implementation, the phased achievements of human settlements were the result of the collaborative work of different functional departments. Therefore, the current decision-making methods, which lack effective feedback, should be improved in the rural human settlement improvement action, and the previous construction should be evaluated to guide the next round and avoid repeated construction and inefficient resource use.

Because villagers are the primary subjects of the human settlement environment, guiding construction to suit their requirements is the only way to achieve more efficient and long-term growth. The disparity between the actual improvement and the needs of villagers can serve as a guide for future policy optimization in rural human settlement.

### 5.3. Limitations and Further Research

The villagers’ satisfaction evaluation model established on the basis of empirical data analysis was primarily focused on villagers’ needs. This study was an exploration of the methodology used to evaluate subjective satisfaction on the basis of first-hand data. Furthermore, due to the complexity of rural human settlements as a comprehensive system, the analysis method for extracting common factors can be optimized so that it can more comprehensively represent the improvement content, and the interpretation ability of the extracted data can be further strengthened. Improving the evaluation basis of the satisfaction evaluation model will enable a more comprehensive reflection of the real needs of the villagers, and the collection of dynamic data should be continued at different stages to provide efficient feedback for human settlement construction. Future research should emphasize long-term follow-up investigations. Lastly, it was found that the daily high mobility of villagers and the rapid change in rural lifestyles in survey areas pose new challenges to the research of human settlements.

## 6. Conclusions

Rural human settlements are complex organisms, and villagers are the inner determinants. Therefore, the direct purpose of rural human settlement construction is to meet the needs of villagers, and positive coordination between the latest round of construction and the demands of the villagers can ensure the sustainable development of human settlements. This study applied empirical research to find the mismatch between the improvement activities and the villagers’ demands by establishing a satisfaction evaluation system from a local-based and villager-centered perspective, which included living supporting facilities, nonagricultural industry income, agriculture production income, transportation infrastructure, and a comprehensive ecological improvement factor according to weight. This method including a quantitative analysis of satisfaction and quadrant guidance can provide a basis for subsequent programs and settlement improvement policies based on the will of villagers.

According to the analysis at the village level, evaluation results showed that construction investment is useful in enhancing satisfaction, but the level of investment is not directly proportional to satisfaction, and the actual progress cannot keep up with the villagers’ demand for public fitness, cultural, and recreational facilities. In contrast, the changing lifestyles of villagers can reduce the need for commercial facilities. Furthermore, the satisfaction differences generated by village improvement express the villagers’ demand for fairness on a microscale. The selected indicators in the system could be divided into four quadrants according to the combination of importance and satisfaction score. This study can help policymakers reflect on the fit between the current construction and actual villagers’ needs. Although there are significant differences in rural areas of the world, the construction experience and input from rapid urbanization areas can be generally applied to other regions facing great changes under the background of globalization. Constructing rural human settlements is a complicated social practice, and long-term follow-up research on village satisfaction should be pursued in the future to deepen and widen the connotation of rural human settlements.

## Figures and Tables

**Table 1:** Information about the survey area and questionnaire.

Administrative Village	Natural Village	Questionnaire Code	Basic Information		Questionnaire Information		
			Number of Households	Resident Population	Number of Questionnaires	Number of Valid Questionnaires	Valid Rate
Huqiao Village	Huqiaojie	HQJ	48	175	33	30	90.91%
	Hougang	HG	76	300	39	36	92.31%
	xiangnanjiao	XNJ	42	130	20	20	100%
	Dongshiqian	DSQ	48	176	27	24	88.89%
	Zhongshiqian	ZSQ	43	155	16	15	93.75%
Subtotal			257	936	135	125	92.59%
Linghu Village	Dongtang	DT	93	428	35	32	91.43%
	Wengjiabang	WJB	84	275	30	24	80%
	Shuilushang	SLS	155	415	29	29	100%
	Wushe	WS	225	922	48	39	81.25%
	Lubuzhuang	LBZ	115	543	32	31	96.88%
	Xitang	XT	107	277	31	28	90.32%
	Huangshu	HS	75	271	36	30	83.33%
Subtotal			854	3131	241	213	88.38%
Shishe Village	Houfangtang	HFT	44	156	24	21	87.50%
	Lujiabang	LJB	45	180	30	26	86.67%
	Shijiaopen	SJP	45	156	24	19	79.17%
	Bangguojiao	BGJ	62	233	30	28	93.33%
	Zhangjiatang	ZJT	32	123	16	15	93.75%
	Majiajiao	MJJ	23	89	14	11	78.57%
	Beicheng	BC	47	158	27	23	85.19%
	Guanyintang	GYT	35	121	18	15	83.33%
Subtotal			333	1216	183	158	86.34%
Total			1444	5283	521	496	95.20%

**Table 2:** Index system of theoretical model of satisfaction evaluation and common factor variance ratio.

Target	Elements	Indicators	Criteria
Rural human settlement	A. Infrastructure	Perfection and quality of village road regulation (A1)	The Likert scale was used to assign a score ranging from 1 to 5, with 1 being very dissatisfied and 5 being very satisfied.
		Street lighting works (A2)	
		Motor vehicle parking (A3)	
		Riverbank repair including access bridge (A4)	
		Construction of public toilets (A5)	
	B. Quality of living environment	Façade paint of houses and beautification of the head of courtyard wall (B1)	
		Transformation of the microenvironment in front of and behind the house (B2)	
		Public activity site and landscape reconstruction (B3)	
		Village architectural scene beautification (B4)	
		Impact of the industrial development of the village on the living environment (B5)	
	C. Public services	Public fitness facilities (C1)	
		Cultural and recreational facilities (C2)	
		Administrative office facilities (C3)	
		Educational facilities (training exchanges, schools, societies) (C4)	
		Convenient commercial facilities (C5)	
		External public transport (C6)	
	D. Living standards	Improvement of planting conditions and auxiliary facilities in agriculture (D1)	
		Impact of agricultural planting on income (D2)	
		Impact of village collective enterprises on income (D3)	
		Impact of rural tourism development on income (D4)	
		Impact of rural emerging industry development on income (D5)	
	E. Environmental health	Satisfaction with production pollution control level (E1)	
		Satisfaction with domestic waste pollution control level (E2)	
		Satisfaction with water quality in the river (E3)	
		Satisfaction with air quality (E4)	

**Table 3:** Reliability and validity test statistics. Number of variables = 16, *n* = 496.

**Cronbach’s α**		0.868
**Kaiser–Meyer–Olkin Measure**		0.882
Bartlett sphericity test	Approximate chi-square	2791.127
	df	120
	Sig.	0.000

## Data Availability

Not applicable.

## Appendix A

**Table A1:** Common factor variance ratio (number of variables = 16, number of valid questionnaires = 496).

Number	Indicators	Initial	Extraction
X1	Perfection and quality of village road regulation (A1)	1.000	0.612
X2	Motor vehicle parking (A3)	1.000	0.681
X3	External public transport (C6)	1.000	0.635
X4	Construction of public toilets (A5)	1.000	0.519
X5	Public activity site and landscape reconstruction (B3)	1.000	0.650
X6	Public fitness facilities (C1)	1.000	0.699
X7	Cultural and recreational facilities (C2)	1.000	0.677
X8	Educational facilities (training exchanges, schools, societies) (C4)	1.000	0.564
X9	Improvement of planting conditions and auxiliary facilities in agricultural (D1)	1.000	0.733
X10	Impact of agricultural planting on income (D2)	1.000	0.752
X11	Impact of village collective enterprises on income (D3)	1.000	0.584
X12	Impact of rural tourism development on income (D4)	1.000	0.791
X13	Impact of the industrial development of the village on the living environment (B5)	1.000	0.724
X14	Impact of rural emerging industries development on income (D5)	1.000	0.634
X15	Satisfaction with domestic waste pollution control level (E2)	1.000	0.643
X16	Satisfaction with air quality (E4)	1.000	0.745

The extraction method was principal component analysis. The number of common factors after extraction was 5.

**Table A2:** Total variance explained (number of variables = 16, number of valid questionnaires = 496).

Component	Initial Eigenvalues			Extract Quadratic Sum of the Loads			Rotate Quadratic Sum of the Loads		
	Total	Variance Percentage	Accumulative Total %	Total	Variance Percentage	Accumulative Total %	Total	Variance Percentage	Accumulative Total %
1	5.683	35.521	35.521	5.683	35.521	35.521	2.749	17.179	17.179
2	1.545	9.655	45.176	1.545	9.655	45.176	2.496	15.598	32.777
3	1.188	7.426	52.603	1.188	7.426	52.603	2.064	12.899	45.676
4	1.162	7.263	59.866	1.162	7.263	59.866	1.812	11.323	56.999
5	1.064	6.650	66.516	1.064	6.650	66.516	1.523	9.517	66.516
6	0.672	4.201	70.717						
7	0.631	3.941	74.658						
8	0.594	3.710	78.367						
9	0.572	3.574	81.941						
10	0.515	3.216	85.157						
11	0.483	3.019	88.177						
12	0.474	2.966	91.142						
13	0.397	2.483	93.626						
14	0.388	2.426	96.051						
15	0.356	2.226	98.277						
16	0.276	1.723	100.000						

Extraction method: principal component analysis.

**Table A3:** Rotated component loading matrix.

Indicators	Component				
	F1	F2	F3	F4	F5
X1	—	—	—	0.675	—
X2	—	—	—	0.773	—
X3	—	—	—	0.650	—
X4	0.581	—	—	—	—
X5	0.711	—	—	—	—
X6	0.790	—	—	—	—
X7	0.755	—	—	—	—
X8	0.466	—	—	—	—
X9	—	—	0.834	—	—
X10	—	—	0.836	—	—
X11	—	0.455	—	—	—
X12	—	0.841	—	—	—
X13	—	0.780	—	—	—
X14	—	0.647	—	—	—
X15	—	—	—	—	0.729
X16	—	—	—	—	0.842

Values below 0.45 are hidden. Extraction method: principal component analysis. Rotation method: Kaiser normalized maximum variance method. Rotation converged after six iterations.

**Table A4:** Rotated component score coefficient matrix.

Indicators	Component				
	F1	F2	F3	F4	F5
X1	−0.058	−0.072	−0.031	0.426	0.105
X2	−0.040	−0.124	−0.064	0.527	0.022
X3	−0.166	0.095	0.057	0.453	−0.240
X4	0.254	0.085	−0.164	−0.095	0.032
X5	0.341	−0.157	−0.067	0.079	−0.023
X6	0.422	−0.169	−0.044	−0.029	−0.063
X7	0.381	−0.057	−0.023	−0.133	−0.060
X8	0.145	0.087	0.116	−0.048	−0.177
X9	−0.162	−0.045	0.495	0.006	0.043
X10	−0.028	−0.174	0.493	0.012	0.025
X11	0.080	0.134	0.171	−0.209	0.046
X12	−0.117	0.472	−0.148	−0.018	−0.013
X13	−0.155	0.424	−0.112	0.066	−0.001
X14	−0.048	0.297	0.107	−0.132	−0.014
X15	−0.008	−0.111	0.091	−0.029	0.516
X16	−0.133	0.035	−0.041	−0.037	0.619

The extraction method was principal component analysis. Rotation method: Kaiser normalized maximum variance method. Score calculation method: regression method.
